# Mathematical Modeling and Simulations for Large-Strain J-Shaped Diagrams of Soft Biological Materials

**DOI:** 10.3390/polym10070715

**Published:** 2018-06-29

**Authors:** Kazuhiko Mitsuhashi, Swapan Ghosh, Hiroshi Koibuchi

**Affiliations:** Department of Industrial Engineering, National Institute of Technology, Ibaraki College, Nakane 866, Hitachinaka, Ibaraki 312-8508, Japan; kmitsuha@ge.ibaraki-ct.ac.jp (K.M.); skghosh@ge.ibaraki-ct.ac.jp (S.G.)

**Keywords:** soft biological materials, stress–strain diagram, J-shaped diagram, Monte Carlo, statistical mechanics, Finsler geometry

## Abstract

Herein, we study stress–strain diagrams of soft biological materials such as animal skin, muscles, and arteries by Finsler geometry (FG) modeling. The stress–strain diagram of these biological materials is always J-shaped and is composed of toe, heel, linear, and failure regions. In the toe region, the stress is almost zero, and the length of this zero-stress region becomes very large (≃150%) in, for example, certain arteries. In this paper, we study long-toe diagrams using two-dimensional (2D) and 3D FG modeling techniques and Monte Carlo (MC) simulations. We find that, except for the failure region, large-strain J-shaped diagrams are successfully reproduced by the FG models. This implies that the complex J-shaped curves originate from the interaction between the directional and positional degrees of freedom of polymeric molecules, as implemented in the FG model.

## 1. Introduction

Biological materials such as muscles, tendons, and skin are known to be very flexible and strong, and for this reason, these materials have attracted considerable interest with regard to the design of artificial materials or meta-materials [1,2]. The mechanical properties of these materials are of fundamental importance in their applications [3,4]. The stress–strain diagram is a typical approach for characterizing the mechanical strength of these materials, and numerous experimental studies on this topic have been conducted [5,6,7,8,9,10,11,12,13,14,15,16,17,18,19].

**Figure 1:**
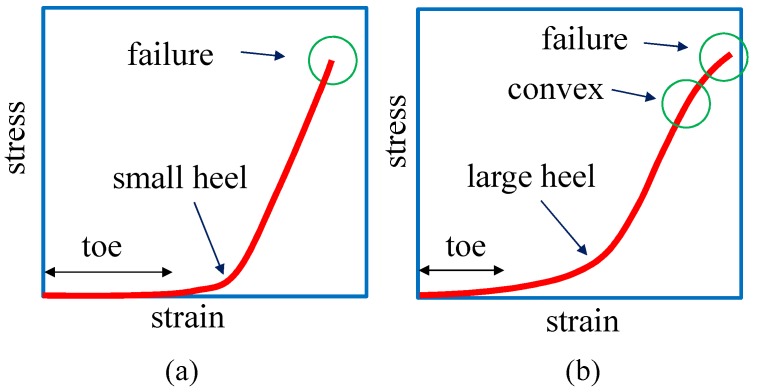
A J-shaped diagram is composed of two different linear lines, and the region where two lines are smoothly connected is called the heel. The J-shaped diagrams are decomposed into two groups: the diagrams with (**a**) a small heel and (**b**) a large heel. The curve in (**b**) is convex upward in the large-strain region and hence is highly non-linear.

It has been experimentally observed that the stress–strain diagram of soft biological materials is J-shaped and that the curve is composed of toe, heel, linear and rupture (or failure) regions (Figure 1a,b) [5,6,7,8,9,10,11,12,13,14,15,16,17,18,19]. The failure region is beyond the scope of this paper and will not be taken into consideration. In the toe region, the stress is almost zero, implying that the materials freely extend without external forces, similar to the behavior of the soft-elasticity region of liquid-crystal elastomers [20,21,22,23,24,25,26]. The existence of this zero-stress region is the reason why we call the curve J-shaped.

The continuum mechanics approach based on the strain energy functional successfully describes the J-shaped diagrams [7]. In the context of continuum mechanics, the non-linearity in the J-shaped curve is understood as hyperelasticity [27,28]. However, the full information of the position of polymer is not always included in those modelings. In fact, the strains are used to obtain the diagram in those models, and the strains are calculated from the displacement field u, which is a part of the position variable r of polymers such that $r=r_{0}+u$. This convention is useful if u is very small compared to $r_{0}$, and it is used in continuum mechanics or elasticity theory. If both $r_{0}$ and u are obtained in this expression, we can evaluate r [29,30]. However, it is not necessary to separate r into two parts in the case of polymers, and the polymer position r is more convenient than the strain. Indeed, an interaction of the polymer position and direction is defined by using r and σ and implemented in the FG model, as we will see in the following section. As a result of this interaction, shape dependent mechanical property and its relation to σ can be obtained independently of how external stimuli are given. To calculate the stress for example, we impose a constraint on the strain by fixing the polymer position r of the boundary. This constraint induces an alignment of σ, and the induced internal structural change of σ causes a nontrivial behavior of mechanical property such as J-shaped diagram. Such a qualitative understanding for biological materials is actually observed in the FG model. For this reason, studying the diagram using the Finsler geometry (FG) modeling technique, where the polymer position r is directly used, is an interesting prospect.

We have proposed the FG modeling technique to study anisotropic phenomena such as liquid-crystal elastomers elongation and soft elasticity [31,32]. In [33,34], we studied J-shaped curves by this FG modeling technique and obtained Monte Carlo (MC) data consistent with previously reported experimental results, in which the toe length is up to 40∼50% on the strain axis. For these experimental J-shaped curves of small toe length, FG modeling successfully describes the diagrams.

However, the toe length reaches 150% in some biological materials [9]. The length of the toe region is generally, albeit not always, limited to less than 50% [5,6,7,8,9]. The main component maintaining the mechanical strength is the collagen fibers, and elastin also plays an important role in the mechanical property [1]. Moreover, a lot of components in those biological materials are expected to contribute to the mechanical strength of materials with large toe length. Thus, it is very difficult to study the diagram by incorporating these complex mechanisms. Therefore, it is worthwhile to use the FG modeling technique for studying the large strain diagram.

In this paper, the existing experimental J-shape curves of biological materials such as animal skin, muscles, and arteries are compared with the simulation results. The experimental curves of these materials are grouped into two types: the group of diagrams with a small heel (S-heel) and the group of diagrams with a large heel (L-heel) (Figure 1a,b) [6,7,8,9]. The diagram with an S-heel (Figure 1a) is decomposed into two straight lines with different slopes, while the diagram with an L-heel is decomposed into two different linear lines and a curve for the heel between the two lines. Moreover, close to the failure region, some of the curves have a convex part, which seems to correspond to the failure region where the collagen fibers start to break (Figure 1b).

## 2. Models and Monte Carlo Simulations

**Figure 2:**
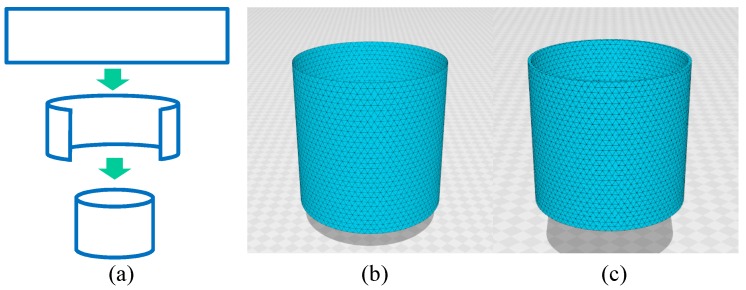
(**a**) A rectangular surface forms a cylindrical surface. Cylindrical lattices for (**b**) 2D and (**c**) 3D FG models. The lattices are composed of (**b**) triangles and (**c**) tetrahedrons. The total number of vertices are (**b**) N=2511 and (**c**) N=5022.

We use cylindrical surfaces that are suitable for the calculation of the surface tension or the stress. The cylindrical surface is obtained by bending and identifying two boundaries opposite to each other of a rectangular surface (Figure 2a). The remaining two boundaries correspond to the boundaries of the cylinder, which are fixed to calculate the surface tension. Since the simulations are performed on the lattices of finite size, it is better to remove unexpected boundary effects in the simulations of surface tension. For this reason, the surface boundaries, which are not directly connected to the surface tension calculation, should be removed.

**Figure 3:**
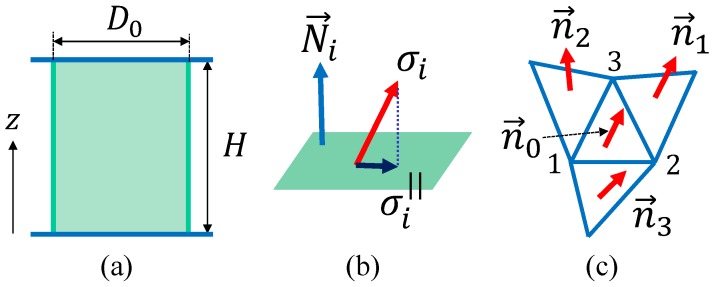
(**a**) The height *H* and diameter $D_{0}$ of a cylinder, (**b**) the unit normal vector $N_{i}$ of the tangential plane at the vertex *i*, the tangential component $\sigma_{i}^{||}$ of the variable $\sigma_{i}$, and (**c**) the unit normal vectors $n_{i}$ of the triangles i(=0,1,2,3) used in $S_{2}$ of Equation (4). σ is identified with −σ because of the non-polar interaction.

The 2D FG model is defined on a cylindrical lattice like the one in Figure 2b. The size of the lattice in Figure 2b is given by $\left(N,N_{B},N_{T}\right)=\left(2511,7371,4860\right)$, where *N*, $N_{B}$, and $N_{T}$ are the total number of vertices, the total number of bonds, and the total number of triangles, respectively. The Euler number χ is used to check whether the lattice is correctly constructed, and the χ of the cylinder must be the same as that of the torus, so $\chi =N-N_{B}+N_{T}=0$. The lattice in Figure 2c is constructed from tetrahedrons for the 3D FG model [32], and the surfaces inside and outside the 3D lattice are 2D cylinders that are exactly the same as the one in Figure 2b. *Netgen Mesh Generator* is used to generate the 3D lattices for the simulations and in Figure 2c, where the 2D cylinder lattice in Figure 2b is used as the input data. This 3D lattice is thin, so all vertices are on the surface; there are no vertices inside the 3D structure. The lattice size is given by $\left(N,N_{B},N_{T},N_{\mathrm{tet}}\right)$ = (5022, 24624, 34182, 14580), where the first three symbols are the same as those for 2D lattice and $N_{\mathrm{tet}}$ is the total number of tetrahedrons. This 3D lattice is topologically identical to the torus and has zero Euler number $\chi =N-N_{B}+N_{T}-N_{\mathrm{tet}}=0$. The height *H* is fixed during the simulations for the calculation of the tensile force (Figure 3a). The diameter *D* of the upper and lower boundaries is also fixed to $D_{0}$, and this boundary condition protects the cylinder from collapsing for small bending rigidity in the simulations. It should be emphasized that this constraint for *D* on the boundaries is close to the experimental setup for the measurement of tensile force.

Here we should note that the 3D model defined on 3D body such as 3D cylinders is not used here, because the stress–strain diagram does not have J-shaped behaviors at least on the cylinder of which the diameter and height are comparable [32]. As we will see from the simulation results, large heel diagrams are obtained only from the 3D model on thick cylindrical surface (Figure 2c), and diagrams with convex are obtained only from 2D model on cylindrical surface (Figure 2b). It is unclear whether these 2D and 3D models can be applied to an arbitrary J-shaped curve or not. At present, it seems very hard for these models to reproduce curves that are far different from those in Figure 1a,b.

### 2.1. 2D Model

In this subsection, we describe the 2D FG model. Although this 2D model is the same as the one in [33], we summarize the Hamiltonian here in a self-contained manner. The Hamiltonian is given by a linear combination of four terms such that

(1) $$\begin{array}{c} S\left(\sigma ,r\right)=\lambda S_{0}+\gamma S_{1}+\kappa S_{2}+U_{B},\;\left(\gamma =1\right)\\ S_{0}\left(\sigma \right)=-\left(3/2\right)\sum_{ij}{\left(\sigma_{i}^{||}\cdot \sigma_{j}^{||}\right)}^{2}\\ U_{B}=\sum_{i\in \mathrm{boundary}}U_{B}\left(r_{i}\right),\;U_{B}\left(r_{i}\right)=\left\{\begin{array}{ll} \infty & \left(|z_{i}-H|>\delta_{B}\;\mathrm{or}\;|z_{i}|>\delta_{B}\right)\\ 0 & \left(\mathrm{otherwise}\right) \end{array}\right.. \end{array}$$

The variable $r(\in R^{3})$ is the vertex position and represents the position of a polymer, such as collagen fibers. The direction of the polymer is represented by $\sigma (\in S^{2})$, which has a non-polar interaction of the Lebwohl–Lasher type [35] described by $S_{0}$; hence, σ is identified with −σ. We should note that the edges of the triangles do not always represent linear polymers or polymer networks [36]. The triangles are simply introduced for the discretization of 2D materials [37,38,39,40,41,42]. Indeed, the triangle edges play a role as local coordinate axes for the discretization of the Hamiltonian. The variable $\sigma_{i}^{||}$ in $S_{0}$ is defined by

(2) $$\begin{array}{c} \sigma_{i}^{||}=\sigma_{i}-\left(\sigma_{i}\cdot N_{i}\right)N_{i}, \end{array}$$

which is a component of $\sigma_{i}$ parallel to the tangential plane at the vertex *i* (Figure 3b). This tangential plane is determined by its unit normal vector $N_{i}$ (Figure 3b), which is defined such that

(3) $$\begin{array}{c} N_{i}=\frac{\sum_{j(i)}A_{j(i)}n_{j(i)}}{\left|\sum_{j(i)}A_{j(i)}n_{j(i)}\right|} \end{array}$$

where $A_{j(i)}$ and $n_{j(i)}$ denote the area and the unit normal vector of the triangle j(i) sharing the vertex *i*, respectively (Figure 3c).

The Gaussian bond potential $S_{1}$ and the bending energy $S_{2}$ are given by

(4) $$\begin{array}{c} S_{1}=\sum_{\Delta }\left(\gamma_{12}\ell_{12}^{2}+\gamma_{23}\ell_{23}^{2}+\gamma_{31}\ell_{31}^{2}\right),\;\ell_{ij}^{2}={\left(r_{i}-r_{j}\right)}^{2}\\ S_{2}=\sum_{\Delta }\left[\kappa_{12}\left(1-n_{0}\cdot n_{3}\right)+\kappa_{23}\left(1-n_{0}\cdot n_{1}\right)+\kappa_{31}\left(1-n_{0}\cdot n_{2}\right)\right]\\ \gamma_{12}=\frac{1}{6}\left(\frac{v_{12}}{v_{13}}+\frac{v_{21}}{v_{23}}\right),\;\gamma_{23}=\frac{1}{6}\left(\frac{v_{23}}{v_{21}}+\frac{v_{32}}{v_{31}}\right),\;\gamma_{31}=\frac{1}{6}\left(\frac{v_{31}}{v_{32}}+\frac{v_{13}}{v_{12}}\right)\\ \kappa_{12}=\frac{1}{6}\left(\frac{v_{13}}{v_{12}}+\frac{v_{23}}{v_{21}}\right),\;\kappa_{23}=\frac{1}{6}\left(\frac{v_{21}}{v_{23}}+\frac{v_{31}}{v_{32}}\right),\;\kappa_{31}=\frac{1}{6}\left(\frac{v_{32}}{v_{31}}+\frac{v_{12}}{v_{13}}\right). \end{array}$$

In these expressions, $\ell_{ij}$ is the length of bond ij connecting the vertices *i* and *j*, and $n_{i}$ is a unit normal vector of the triangle *i*. The coefficient $\gamma_{ij}$ in $S_{1}$ is defined by using $v_{ij}$, which is given by

(5) $$\begin{array}{c} v_{ij}=\left|\sigma_{i}\cdot t_{ij}\right|,\;t_{ij}=\frac{{\vec{\ell }}_{ij}}{\left|\ell_{ij}\right|},\;{\vec{\ell }}_{ij}=r_{j}-r_{i}. \end{array}$$

(see [32] for more detailed information on the discretization of $S_{1}$ and $S_{2}$). The quantities $\gamma_{ij}\left(=\gamma_{ji}\right)$ and $\kappa_{ij}\left(=\kappa_{ji}\right)$ are considered as the position- and direction-dependent surface tension and bending rigidity, respectively. The surface tension coefficient γ is fixed to γ=1 for simplicity and is not used henceforth.

The potential $U_{B}$ allows the boundary vertices to move vertically in the *z*-direction within a small range $\pm \delta_{B}$, which is fixed to the mean bond length. This constraint does not influence the results in the limit of N→∞ because $\delta_{B}/H$ is negligible in this limit

(6) $$\begin{array}{c} \frac{\delta_{B}}{H}\left(=\frac{\mathrm{mean}\;\mathrm{bond}\;\mathrm{length}}{\mathrm{height}\;\mathrm{of}\;\mathrm{cylinder}}\right)\to 0\;\left(N\to \infty \right), \end{array}$$

since the mean bond length is independent of *N*, while *H* is proportional to *N*. The reason for this constraint $U_{B}$ is assumed to be avoiding a strong and non-physical force, which is suspected to appear when $\sigma_{i}$ aligns with the *z*-direction on the boundary. If $\sigma_{i}$ on the boundary aligns with the *z*-direction without $U_{B}$, the corresponding $v_{ij}$ becomes $v_{ij}\to 0$ because of the definition of $v_{ij}$ in Equation (5). Therefore, the corresponding $\gamma_{jk}$ becomes $\gamma_{jk}\to \infty$ and hence $S_{1}\to \infty$. In this situation, the variable $\sigma_{i}$ never aligns with the *z*-direction, so $U_{B}$ is necessary for the well-definedness of the model.

The partition function is given by

(7) $$\begin{array}{c} Z_{2D}\left(\lambda ,\kappa ;H\right)=\sum_{\sigma }\int \prod_{i=1}^{2N_{1}}dr_{i}\prod_{i=1}^{N-2N_{1}}dr_{i}\exp\left[-S(\sigma ,r)\right] \end{array}$$

where *H* is the height of the cylinder and is fixed during the simulation (Figure 3a). $\int \prod_{i=1}^{2N_{1}}dr_{i}$ denotes the 1D integrations of the vertices on the boundaries, where $N_{1}$ is the total number of vertices on the upper and lower boundaries. The $2N_{1}$ vertices are allowed to move along the circles of radius $D_{0}$, so the corresponding integration effectively becomes one-dimensional. The total number of remaining vertices is $N-2N_{1}$, and the positions of these vertices are integrated out by the 3D integrations represented by $\int \prod_{i=1}^{N-2N_{1}}dr_{i}$.

### 2.2. 3D Model

**Figure 4:**
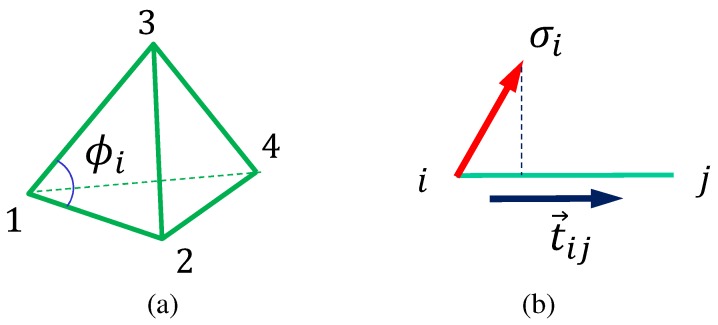
(**a**) A tetrahedron with vertices (1, 2, 3, 4) and an internal angle $\phi_{i}$ of a triangle and (**b**) the variable $\sigma_{i}$ and the unit tangential vector $t_{ij}$ of the bond connecting the vertices *i* and *j*. The $\sigma_{i}$ and $t_{ij}$ are used to define $v_{ij}$ in Equation (5).

The 3D model Hamiltonian is defined on the 3D lattice discretized by the tetrahedrons shown in Figure 4a. Although the Hamiltonian is almost the same as that in [32], we briefly describe it in the outline below. The model is defined without the self-avoiding potential for the “surfaces” (not for the inside of the structure), and this is the only difference between the models in this paper and in [32]. The surface self-avoiding potential is a non-local potential and is time-consuming for simulations. We expect that the results are not strongly influenced by whether this self-avoiding interaction is included or not because the upper and lower boundaries are fixed and the surface always remains relatively smooth. This is also expected for the 2D model, which has no self-avoiding potential.

The Hamiltonian S(r,σ) is defined by a linear combination of five different terms:

(8) $$\begin{array}{c} S\left(r,\sigma \right)=\lambda S_{0}\left(\sigma \right)+S_{1}\left(r,\sigma \right)+\kappa S_{2}\left(r\right)+U_{3D}+U_{B}\\ S_{0}\left(\sigma \right)=\frac{1}{2}\sum_{ij}\left[1-3{\left(\sigma_{i}\cdot \sigma_{j}\right)}^{2}\right]\\ S_{1}=\sum_{ij}\Gamma_{ij}\ell_{ij}^{2},\;\Gamma_{ij}=\frac{1}{4\bar{N}}\sum_{\mathrm{tet}}\gamma_{ij}\left(\mathrm{tet}\right)\\ S_{2}\left(r\right)=\sum_{i}\left[1-\cos(\phi_{i}-\pi /3)\right]\\ U_{3D}=\sum_{\mathrm{tet}}U_{3D}\left(\mathrm{tet}\right),\;U_{3D}\left(\mathrm{tet}\right)=\left\{\begin{array}{ll} 0 & \left(\mathrm{Vol}(\mathrm{tet})>0\right)\\ \infty & \left(\mathrm{otherwise}\right) \end{array}\right.. \end{array}$$

The variable $r(\in R^{3})$ is the vertex position of a tetrahedron, and $\sigma (\in S^{2})$ denotes the directional degrees of freedom of polymers exactly the same as in the 2D model. Each term shares the same role with the corresponding term in the 2D model. The definition of the Lebwohl–Lasher potential $S_{0}$ is slightly different from that of $S_{0}$ in Equation (1), but the role of this term in the 3D model is identical to that of $S_{0}$ in the 2D model. The definition of $S_{1}$ is also slightly different from the 2D case; however, the continuous description of $S_{1}$ is the same (see [32]). In the coefficient of $\Gamma_{ij}$, $\bar{N}$ is defined by

(9) $$\begin{array}{c} \bar{N}=\frac{1}{N_{B}}\sum_{ij}n_{ij} \end{array}$$

where $n_{ij}$ is the total number of tetrahedrons sharing the bond ij and $N_{B}\left(=\sum_{ij}1\right)$ is the total number of bonds. The $\gamma_{ij}\left(\mathrm{tet}\right)$ in $S_{1}$ is given by

(10) $$\begin{array}{c} \gamma_{12}=\frac{v_{12}}{v_{13}v_{14}}+\frac{v_{21}}{v_{23}v_{24}},\;\gamma_{13}=\frac{v_{13}}{v_{12}v_{14}}+\frac{v_{31}}{v_{32}v_{34}},\;\gamma_{14}=\frac{v_{14}}{v_{12}v_{13}}+\frac{v_{41}}{v_{43}v_{42}}\\ \gamma_{23}=\frac{v_{23}}{v_{21}v_{24}}+\frac{v_{32}}{v_{31}v_{34}},\;\gamma_{24}=\frac{v_{24}}{v_{23}v_{21}}+\frac{v_{42}}{v_{41}v_{43}},\;\gamma_{34}=\frac{v_{34}}{v_{31}v_{32}}+\frac{v_{43}}{v_{41}v_{42}} \end{array}$$

where the numbers 1,2,3,4 denote the vertices of the tetrahedron in Figure 4a. In these expressions for $\gamma_{ij}$, the symbol $v_{ij}$ is defined by the same expression as in Equation (5) using $\sigma_{i}$ and the unit tangential vector $t_{ij}$ along the tetrahedron edge ij (Figure 4b).

The term $S_{2}$ in Equation (8) is different from that of the 2D model in Equation (1); however, the role of $S_{2}$ in Equation (8), i.e., to keep the tetrahedron shape almost regular for positive κ values, is the same as that in the 2D model. The symbol $\phi_{i}$ is the internal angle of triangles (Figure 4a). The role of the potential $U_{3D}$ is to protect the tetrahedron volume from being negative. This potential $U_{3D}$ introduces a repulsive interaction between the vertices so that the tetrahedron is hardly collapsed, so this $U_{3D}$ shares the same role with $S_{2}$ in part. For this reason, the tetrahedron hardly deforms for positive κ values, so we assume small negative κ values in the simulations to give the J-shaped diagrams a large strain. The potential $U_{B}$ is exactly the same as in Equation (1) for the 2D model, and for this reason, its definition is not written in Equation (8).

The partition function $Z_{3D}$ can also be defined for the 3D model; however, its description is exactly the same as $Z_{2D}$ in Equation (7) except for the actual number $N_{1}$ for the boundary vertices. To avoid redundancy, it is not written here.

### 2.3. Formula for Stress Calculation

In both the 2D and 3D models, the stress in the stress–strain diagram is calculated from the principle of scale invariance of the partition function ${\left.dZ/d\alpha \right|}_{\alpha =1}=0$ (for simplicity, the subscript 2D in $Z_{2D}$ is not written henceforth) [43]. This invariance simply originates from the fact that the integrations in *Z* are independent of its expression for r, the position of the material.

If we change r to αr with a positive number α, we have the scaled partition function such that

(11) $$\begin{array}{c} Z\left(\alpha ;A_{p}\left(\alpha \right)\right)=\alpha^{3N-4N_{1}}\sum_{\sigma }\int \prod_{i=1}^{2N_{1}}dr_{i}\prod_{i=1}^{N-2N_{1}}dr_{i}\exp\left[-S(\sigma ,\alpha r)\right], \end{array}$$

where $A_{p}$ is the projected area of the surface and $N_{1}$ is the total number of boundary vertices, as mentioned in the previous subsection. The expression $Z(\alpha ;A_{p}\left(\alpha \right))$ implies that *Z* depends both explicitly and implicitly on α. In the Hamiltonian S(σ,αr), the only term that depends on α is $S_{1}$: $S_{1}\left(\alpha \right)=\alpha^{2}S_{1}$. We should note that the coefficient $\alpha^{3N-4N_{1}}$ in the right hand side of Equation (11) comes from the 3D and 1D integrations such that $\alpha^{3N-4N_{1}}=\alpha^{3(N-2N_{1})}\alpha^{2N_{1}}$.

From the abovementioned scale invariance of *Z*, we have ${\left.d\log Z/d\alpha \right|}_{\alpha =1}=0$ and

(12) $$\begin{array}{c} 3N-4N_{1}-2\gamma \langle S_{1}\rangle -2\frac{A_{p}}{Z}\frac{\partial Z}{\partial A_{p}}=0. \end{array}$$

**Figure 5:**
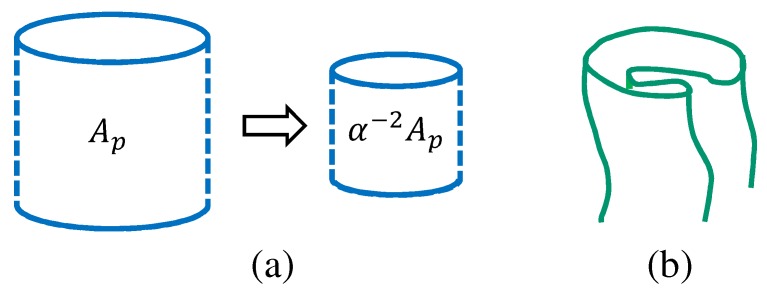
(**a**) An illustration of the change in the projected area $\alpha^{-2}A_{p}$, which restores the original $A_{p}$ and remains unchanged under the scale change r→αr in the partition function, and (**b**) a possible folding of the surface (which is magnified; this type of folding is always suppressed).

For the last term on the left hand side, we assume that $A_{p}\left(\alpha \right)=\alpha^{-2}A_{p}$ for the dependence of $A_{p}\left(\alpha \right)$ on α because the projected area $A_{p}$ is kept fixed under the scale change r→αr for the evaluation of the tensile stress τ (Figure 5a). Moreover, to evaluate $\partial Z/\partial A_{p}$ on the left hand side of Equation (12), we naturally assume that the surface is sufficiently expanded. Under this condition, the free energy *F* of the surface is given by

(13) $$\begin{array}{c} F=\tau \int_{A_{0}}^{A_{p}}dA=\tau \left(A_{p}-A_{0}\right) \end{array}$$

where $A_{0}$ is the area of the surface corresponding to the zero-tensile force [43]. Thus, we have the partition function Z=exp(−F). Inserting this *Z* into Equation (12), we have

(14) $$\begin{array}{c} \tau =\frac{2\langle S_{1}\rangle -3N+4N_{1}}{2A_{p}},\;A_{p}=\pi D_{0}H \end{array}$$

where $D_{0}$ is the diameter of the boundary. We should note that $A_{0}=\pi D_{0}^{2}$, where the initial height is given by $H=D_{0}$. This surface tension τ in Equation (14) is called the frame tension because τ depends only on the area of the frame on which the surface spans. We should note that the formula for τ of the 3D model is the same as Equation (14) for the 2D model. This is because the thickness of the 3D cylinder the for 3D model shown in Figure 2c is sufficiently thin that this 3D cylinder is regarded as a 2D surface.

The reason why $D_{0}=H$ is assumed in the configurations for τ=0 is because the lattice is constructed under the condition $D_{0}=H$ with a regular triangle (see Figure 2b,c). The edge length is expected to be uniform and independent of the direction in the initial undeformed configuration if $D_{0}=H$ is satisfied, at least for λ→0. Therefore, we have no reason to fix $D_{0}$, for example, $D_{0}\neq H$, although a non-zero λ is assumed in both the 2D and 3D simulations.

### 2.4. Comparison with Experimental Data

To compare the simulation result τ in Equation (14) with the experimental data, we have to change simulation units to physical units. For this purpose, we explicitly use $k_{B}T$ and the lattice spacing *a* [33,44], which are suppressed by $k_{B}T=1$ and a=1 in the expression for τ in Equation (14). All quantities that have units of length are multiplied by *a*, and the Boltzmann factor exp(−F) is replaced by exp(−βF), where $\beta =1/k_{B}T$. It should also be noted that τ is the surface tension and has units of [N/m], whereas the experimentally measured stress has units of $\left[N/m^{2}\right]$. Because of this difference in the units, the simulation data τ should be divided by *a* when comparing them to the experimental data. Thus, we have the expression $\tau_{\mathrm{sim}}$ for the simulation data with units of $\left[N/m^{2}\right]$:

(15) $$\begin{array}{c} \tau_{\mathrm{sim}}=\frac{k_{B}T}{a^{3}}\tau =\left(\frac{4\times 10^{-21}}{a^{3}}\right)\tau \;\left[N/m^{2}\right]. \end{array}$$

In this expression, *a* is varied to modify the simulation data τ, and the modified $\tau_{\mathrm{sim}}$ can be compared to the experimentally observed stress $\tau_{\exp}$. The detailed information of *a* will be presented for each $\tau_{\exp}$ in the presentation section.

The Young’s modulus *E* can also be determined from the linear region of the simulation data τ by dividing τ by the strain. The obtained *E* is modified by the same factor in $\tau_{\mathrm{sim}}$ such that

(16) $$\begin{array}{ccc} E_{\mathrm{sim}} & = & \left(\frac{4\times 10^{-21}}{a^{3}}\right)E\;\left[N/m^{2}\right]. \end{array}$$

This $E_{\mathrm{sim}}$ is directly compared to the experimental Young’s modulus $E_{\exp}$, which is called the stiffness and determined from the linear region of $\tau_{\exp}$ [5,8,9,10]. The value of *a* in this $E_{\mathrm{sim}}$ is the same as that of *a* in $\tau_{\mathrm{sim}}$. Therefore, $E_{\exp}$ is not independent of $\tau_{\exp}$. For this reason, we compare only $\tau_{\exp}$ with $\tau_{\mathrm{sim}}$.

We should note that, among the parameters used in the simulation, not all of them are always comparable to physical quantities. The only quantities that can be compared to the experimental ones are $\tau_{\mathrm{sim}}$ and $E_{\mathrm{sim}}$. In fact, we assume that κ is negative in the 3D FG model. The reason for the negative κ is that the tetrahedrons hardly deform for positive κ values, i.e., where the obtained diagram has no toe region, as mentioned above. More detailed information on this negative κ will be described in the presentation section.

### 2.5. Monte Carlo Technique

The standard Metropolis MC technique is used to update the variables r and σ [45,46]. The variable σ is updated by using three different uniform random numbers, and the new variable $\sigma^{\prime }$ is defined independently of the old σ. The variable r is updated such that $r\to r^{\prime }=r+\delta r$ with a small random vector δr. This vector δr is randomly generated in a sphere of radius $d_{0}$, which is fixed for an approximately 50% acceptance rate.

On the upper and lower boundaries, the new position $r^{\prime }$ is constrained such that the diameter of the boundaries remains constant at $D_{0}$, as mentioned above. For the 3D model, two different diameters, $D_{0}^{\mathrm{in}}$ and $D_{0}^{\mathrm{out}}\left(=D_{0}\right)$, are assumed: one for the inner cylinder and the other for the outer one. The difference is given by $D_{0}^{\mathrm{out}}-D_{0}^{\mathrm{in}}=\sqrt{3}\langle \ell \rangle$, where 〈ℓ〉 is the mean bond length of the initial configuration for the simulation. In the discussions below, we use only $D_{0}$ for simplicity. The constraint on the diameter allows the vertices to move only along the circles of diameter $D_{0}$. Due to this free movement of vertices along the circle, it is possible that the surface will become folded for a range of relatively small κ values when the height *H* is sufficiently small and close to $D_{0}$ (Figure 5b). However, as will be seen in the snapshots of the surfaces, no folding is expected.

Another constraint is imposed on the boundary vertices by $U_{B}$ in Equation (1). Under this $U_{B}$, the vertex positions can move in the vertical direction (⇔*z*-direction) within the small range $\delta_{B}$. This $\delta_{B}$ is fixed to the mean bond length, as described in the Section 2.1.

The lattice size for 2D simulations is $\left(N,N_{B},N_{P}\right)$ = (10584, 31416, 20832), and the size for 3D simulations is $\left(N,N_{B},N_{T},N_{\mathrm{tet}}\right)$ = (9761, 48124, 66965, 28602). For this 3D lattice, which is constructed using the same technique as the lattice in Figure 2c, there are no vertices inside the structure, and all the vertices are on the surface.

## 3. Simulation Results

### 3.1. Comparison with Experimental Data

**Figure 6:**
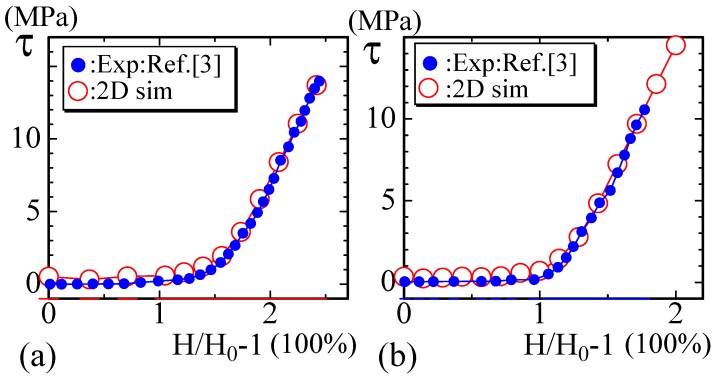
Experimental data $\tau_{\exp}$ (Exp) and the simulation data $\tau_{\mathrm{sim}}$ (sim) for snake skin with a toe region of (**a**) 125% and (**b**) 100% in units of strain [5]. These experimental data are examples of diagrams with an S-heel, and the 2D simulation results are used for comparison with these experimental data.

We show in Figure 6 the experimental stress–strain data of snake’s skin reported in [5]. The skin of snakes, which is composed of collagen fibers and elastin, has a relatively large deformation, as expected from their typical body elongation and bending. The units of the stress $\tau_{\exp}$ are [MPa], and the snake skin is relatively strong. The toe region ranges from 50% to 125% depending on the body position from which the skin is sampled. The sampling positions of the data (Exp) in Figure 6a,b are 40% and 60% distant from the snake’s snout, where 100% corresponds to the length between the snout and the vent. The toe length of the plotted data in Figure 6a,b is relatively large and almost equal to 100% and 125% in units of strain. These curves are typical examples of diagrams with an S-heel, as mentioned in the Introduction, and they are composed of two different linear lines. The parameters used for the simulations and the value of *a* for the fitting of $\tau_{\mathrm{sim}}$ in Equation (15) are summarized below in Table 1.

The stress $\tau_{\mathrm{sim}}$ in the 2D model in Figure 6a is calculated by Equation (15) using the simulation data τ, and $\tau_{\mathrm{sim}}$ is found to be almost identical to the experimental data $\tau_{\exp}$. The parameter λ in Equation (1) is fixed to λ=1, and the bending rigidity κ is varied for the simulations of the 2D model in this paper. The assumed bending rigidities are κ=0.6 and κ=0.55 for the 2D simulations in Figure 6a,b, respectively. The lattice spacing *a* in Equation (15) used for the fitting is $a=0.83\times 10^{-9}$ in Figure 6a and $a=0.85\times 10^{-9}$ in Figure 6b, both of which are larger than the Van der Waals radius (∼1 × $10^{-10}$ [m]), i.e., the typical size of atoms. This is the reason why we call the FG model a coarse-grained model.

We should comment on the reason why the simulation data at the toe region are slightly larger than the EXP data in Figure 6a,b. One of the reasons for the deviation comes from an error in the simulations, because the factor $k_{B}T/a^{3}$ in Equation (15), which is multiplied to the simulation result τ, is of the order $10^{5}$, which is very large. For this reason, a small error in τ at the toe region is magnified. In fact, to make τ=0 at $H/H_{0}=1$, we should carefully find $H_{0}$ with suitable parameters κ and λ. This $H_{0}$ is actually not so easy to find especially for the large $\tau_{\exp}$ cases such as those in Figure 6a,b.

**Figure 7:**
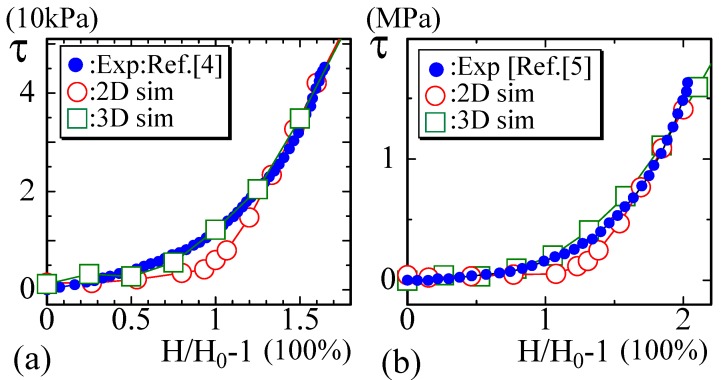
The experimental diagrams of soft biological materials such as (**a**) skin and (**b**) arteries reported in [6,7]. These experimental data are examples of diagrams with an L-heel, and the 2D simulation data slightly deviate from the experimental data in both (**a**) and (**b**).

The second and third sets of experimental data are of soft biological materials such as diaphragm and arteries, to which the 2D model data are not always well fitted. The data plotted in Figure 7a are those obtained in the study of the mechanical diaphragm in a model of muscular dystrophy [6]. The data in Figure 7b are the diagram measured along the circumferential axis of arteries [7]. In [7], Arroyave et al. analyzed the experimental data by using continuous mechanical models (Fung’s Model and Holzapfel’s Model). It was found that abdominal aortas can support higher stress before rupture due to the presence of collagen in the samples. It is also possible to understand that high percentages of elastin are the reason for the large strain in all groups. We should emphasize that the FG modeling technique also successfully reproduces the experimental results, although the experimental data are obtained by using a biaxial loading apparatus [6,7].

The curvature of the heel region in both experimental curves plotted in Figure 7a,b is relatively small compared to that in the experimental data shown in Figure 6a,b. For this reason, the fitting of the simulation data of the 2D FG model is not good for these data, while the 3D simulation data are well fitted except in the failure region.

**Figure 8:**
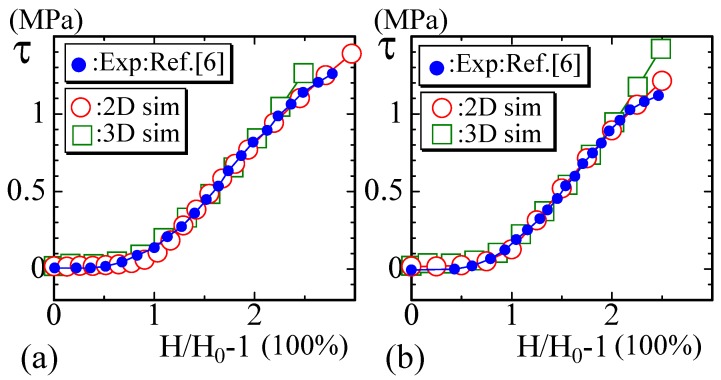
The experimental data are of the periodontal ligament of the molars of rats at (**a**) 6 months and (**b**) 12 months of age [8]. The strain of the toe region is approximately 50%, which is relatively short. For the large-strain region, the experimental data are slightly convex upwards, which is well fitted by the 2D simulation data except in the terminal failure region.

The next experimental data reported in [8] are of the periodontal ligament of the molars of rats at (a) 6 months and (b) 12 months of age (Figure 8a,b. In the case of this material, the strain is over 250% and is larger than that in the previous examples shown in Figure 6 and Figure 7. Moreover, for the large-strain region, the curve of the experimental data starts to bend and becomes convex upwards. The simulation data of 2D model are well fitted even for the convex part (except the failure region), although the 3D simulation data start to deviate from the experimental data in the convex region in Figure 8a. We should emphasize that only the 2D FG model produces results that are in good agreement with the experimental data. In fact, the results of the canonical model are always linear for the large-strain region, and they cannot be fit to the experimental curve with the convex part. This is a non-trivial difference between the FG model and the canonical surface model, as is the fact that the diagram of the canonical surface model becomes linear at a certain value of κ, while the diagram of the FG model is always J-shaped independent of κ [33].

**Figure 9:**
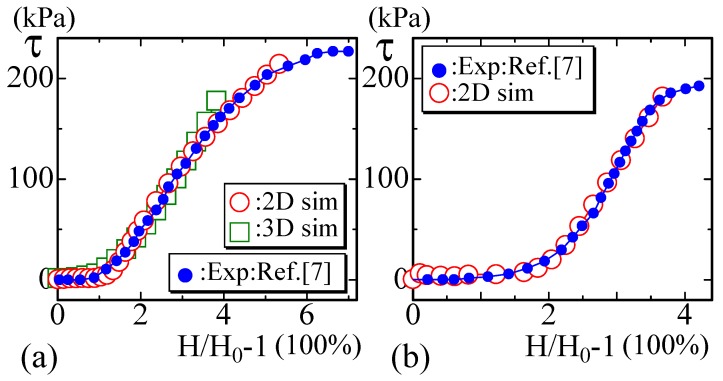
The stress vs. titin strain of animal’s (**a**) cardiac and (**b**) skeletal muscles, where the range of the toe region extends to 100 and 150% [9] and the curves have a convex part in the large-strain region. The strains are very large (up to 700%) compared to those of the other materials studied in this paper.

The final examples of experimental data shown in Figure 9a,b are of the passive tension vs. titin strain of rat muscles, namely, skeletal and cardiac muscles [9]. Granzier et al. studied the mechanical properties of cardiac muscle by investigating passive tension and stiffness in a stretch-and-release process. They estimated the contribution of collagen, titin, microtubules, and intermediate filaments to the tension and the stiffness by gluing experiments after chemical treatments. They concluded that titin and collagen are the best two candidates for explaining the experimental results over a wide range of the lengths. These materials are very soft and flexible, and the titin strain is very large and ranges from 400% to 700%. The experimental curves are convex upward in the large-strain region, and the convex shape is more clear than that in the data shown in Figure 8a,b. In addition to the previous data with the convex part, the results of the 2D FG model better fit the experimental data.

### 3.2. Dependence of the Results on the Simulation Parameters

Here we comment on the dependence of the results on the parameters used in the simulations. The parameters assumed in the simulations are summarized in Table 1. All values of *a* are sufficiently larger than the Van der Waals radius. These are the microscopic parameters and do not always correspond to actual physical quantities, as mentioned in Section 2.4.

First of all, the value of τ (in Figure 6, Figure 7, Figure 8 and Figure 9) is controllable by *a* as described in Section 2.4, while the strain $H/H_{0}-1$ is not because it is dimensionless. Moreover, the behavior of τ in the large strain region is almost linear, and this linear behavior is almost independent of the parameters. For these reasons, the shape of J curve is determined mainly by the length of zero stress region or the plateau between the toe and heel. This plateau length is determined mainly by the initial height $H_{0}\left(=D_{0}\right)$, and it is influenced by λ only slightly. In the simulations, λ is fixed to relatively large values such that the system is in the intermediate phase between the isotropic and aligned phases, where σ locally aligns to a direction spontaneously determined in the cylinders of $H=H_{0}$ for τ=0. This locally aligned direction of σ turns to a uniformly aligned phase along the height direction when the height *H* becomes sufficiently larger than $H_{0}$.

The role of κ in 2D model is to suppress the bending deformation of surfaces, so no effect is expected on τ, at least for sufficiently large *H*, where the surface is smooth along the height direction. In contrast, the fluctuation of surfaces of height $H/H_{0}≃1$ depends on κ. Indeed, the fluctuation of such surfaces $H/H_{0}≃1$ is suppressed if κ is sufficiently large, while the fluctuation is not suppressed if κ is small. This implies that the value of $H_{0}$ itself is strongly dependent on κ, and $H_{0}$ is closely connected to the plateau length. Therefore, κ is crucial to the shape of J curve in the 2D model at least. Indeed, we find from Table 1 that the initial height $H_{0}$ can be fixed in the range $13\leq H_{0}\leq 16$ for κ=0.6. However, when κ decreases to κ=0.55 and κ=0.5, $H_{0}$ also becomes considerably smaller than this range of $H_{0}$.

The stiffness of the 3D model can also be determined by κ, but it also comes from $U_{3D}$, and this is more important in the limit of κ→0. Indeed, the thermal fluctuation of vertices protects the tetrahedron volume from being zero under $U_{3D}$, and as a consequence the volume always remains positive and relatively large even when κ→0. From this effective stiffness, the plateau length becomes very small or almost zero, because no fluctuation is expected in such a 3D cylinder. On such a smooth cylinder, the bond length starts to increase even when *H* is increased only slightly from $H_{0}$, and this leads to an increase of τ. For this reason, κ should be negative. If κ is negative, it is expected that the 3D cylinder starts to fluctuate, and the plateau length then becomes non-zero. For this reason, κ is fixed to small negative such as κ=−0.05 or κ=−0.1.

To summarize, the shape of J curve is determined by the plateau length, and it mainly depends on the stiffness of materials.

### 3.3. Behavior of the Variable σ and Snapshots

To observe how the variable σ aligns, we calculate the order parameter *M* of σ by

(17) $$\begin{array}{c} M=\frac{3}{2}\left(\langle \sigma_{z}^{2}\rangle -\frac{1}{3}\right), \end{array}$$

which represents the alignment of σ along the *z* axis [35]. We also calculate the eigenvalues of the tensor order parameter

(18) $$\begin{array}{c} Q_{\mu \nu }=\frac{3}{2}\left(\langle \sigma_{\mu }\sigma_{\nu }\rangle -\frac{\delta_{\mu \nu }}{3}\right). \end{array}$$

**Figure 10:**
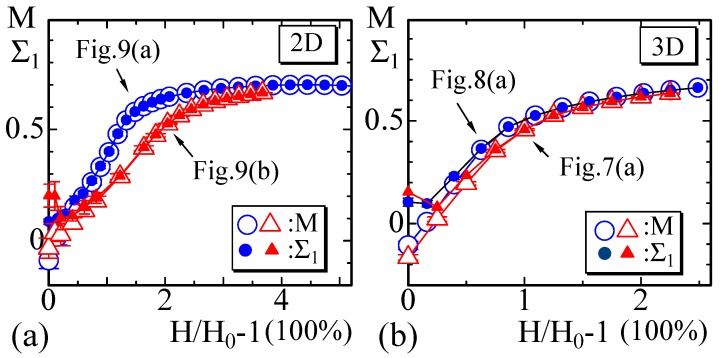
The open symbols in (**a**) 2D and (**b**) 3D simulations represent the order parameter *M* defined by Equation (17), and the solid symbols represent the largest eigenvalue $\Sigma_{1}$ of the tensor order parameter defined by Equation (18).

The largest eigenvalue $\Sigma_{1}$ of $Q_{\mu \nu }$ and *M* corresponding to several simulation results is plotted in Figure 10a,b. For the small-strain region, $\Sigma_{1}$ and *M* slightly deviate from each other; however, they are exactly the same for the large-strain region. This implies that σ aligns in the *z*-direction to which the tensile force is applied.

The problem is what type of configuration of σ appears for the small strain region $H≃H_{0}$, where $\Sigma_{1}$ and *M* are almost identical to each other. To consider this problem, we assume a configuration that σ aligns parallel to the boundary and uniformly encircles the cylinder. It is no wonder that such an anisotropic configuration appears for $H≃H_{0}$, since λ is fixed to relatively large such as λ=1 in the 2D model. If this configuration appears, $\langle \sigma_{z}^{2}\rangle =0$ is expected, and it is also expected that the largest eigenvalue $\Sigma_{1}$ of $Q_{\mu \nu }$ is $\Sigma_{1}=0$. The results in Figure 10a,b show $\Sigma_{1}≃0$ for $H\to H_{0}$ as mentioned above. This indicates the possibility that the configuration of σ is uniformly aligned at $H\to H_{0}$. We should check this with the snapshots below.

**Figure 11:**
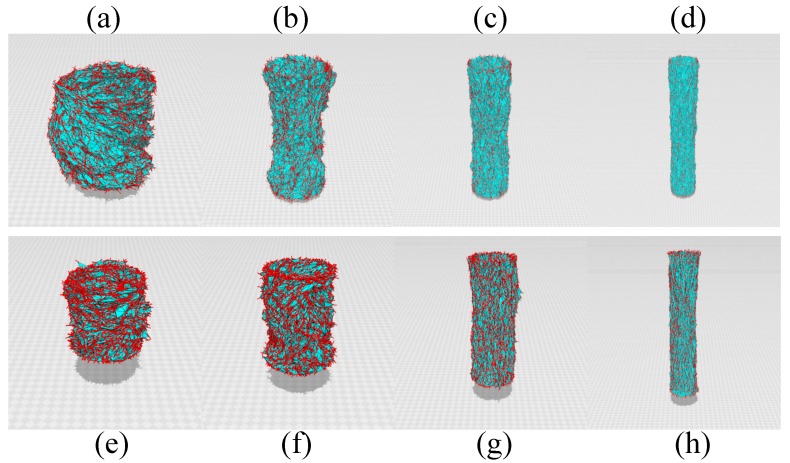
Snapshots of cylindrical surfaces of the 2D model (**a**–**d**) and 3D model (**e**–**h**) corresponding to the simulation data in Figure 9a. The heights of surfaces are (**a**) $H(=D_{0})=14$, (**b**) H=32, (**c**) H=52, and (**d**) H=78 for the 2D model and (**e**) $H(=D_{0})=8.3$, (**f**) H=12, (**g**) H=24, (**h**) H=40 for the 3D model. The scales of the figures are different from each other. The (red) burs on the surface represent the variable σ.

Snapshots of the surfaces are shown in Figure 11a–h, where the scales of the figures are different from each other because the height difference is very large. We find that the variable σ aligns only locally not uniformly at least in the configurations at $H\to H_{0}$ in Figure 11a,e. This implies that σ changes in a similar manner to the directional degree of freedom of polymers, which undergo a transition from a locally ordered polydomain phase to a globally ordered monodomain phase if it is expanded. We should emphasize that the locally ordered configuration is globally random in Figure 11a,e. It is also expected that this locally ordered phase is consistent with the fact that linear objects such as collagen fibers in biological materials are almost aligned even for the released and zero-strain configurations.

## 4. Summary and Conclusions

### 4.1. On the FG Modeling

Finally in this subsection, we should comment on the FG model. In the FG modeling, the geometry inside the materials is modified by replacing the Euclidean metric function by Finsler metric [31,32,33,34], and no interaction energy between polymers is “explicitly” introduced except the Lebwohl–Lasher type potential [35]. As a result of this geometry modification, the interaction between the direction and position of polymers is “implicitly” introduced. For this reason, the FG modeling technique is completely different from the conventional ones, in which an interaction Hamiltonian describing the phenomenon is necessary from the statistical mechanical perspective; to define a model is to introduce an interaction energy in the Hamiltonian. Therefore, for the modeling of complex phenomena, the FG modeling technique has a potential advantage over the ordinary modeling technique, because unknown and complex interaction Hamiltonian is unnecessary.

Hence, the FG modeling technique allows us to study J-shaped diagrams without going into details of the interaction. As a consequence, information on the details of molecular mechanism is hardly obtained. However, it is possible to obtain information on the mechanical properties such as surface tension and bending stiffness of the constituent materials via the assumed parameters γ and κ in Equation (1). The parameter λ is understood as a strength of alignment of polymers, though it is not always considered as a mechanical property. Thus, we consider that information on these mechanical properties of the constituents is useful for a design of new materials, especially in the case of materials, to which a constant response to external mechanical stimuli is requested [3,4].

We should comment on the reason why we use the FG model instead of the canonical surface models. Here we denote the FG model with Euclidean metric by the canonical model. The 2D canonical model is nothing but a surface model for membranes [47,48,49,50,51,52], and here we compare the FG model only with such canonical surface model for membranes or its 3D extended model. The problem is whether or not the results of the canonical 2D and 3D models are identical to those of the FG models. The answer to this question is that the FG model is more suitable than the canonical model. In fact, almost the same results are obtained for some limited range of κ; however, the stress–strain diagram obtained from the canonical 2D model for a certain finite value of κ is not J-shaped, as reported in [33]. Another reason is that only the FG model reproduces the diagrams that are convex upwards for the large-strain region. These are the reasons for why we use the FG modeling technique to analyze the experimental J-shaped diagrams.

In addition to these features of FG modeling, it is important to note that the mechanical strength of real membranes, e.g., the surface tension represented by γ (which is fixed to γ=1 in this paper), becomes dependent on the position and direction on the surface [5,7]. This property is the origin of why the FG model is considered more suitable for actual biological membranes or polymeric sheets than the standard surface models [31]. In the 2D and 3D models, a Finsler metric is assumed in the Gaussian bond potential, and this Finsler metric plays a role in introducing the interaction between the variables σ and r, which correspond to the polymer direction and position, respectively. As a result of this modeling, the mechanical strength, such as the surface tension, effectively depends not only on the position but also on the direction inside the material. Indeed, the Gaussian bond potential $S_{1}=\sum_{ij}\ell_{ij}^{2}$ of the canonical model is changed to $S_{1}=\sum_{ij}\gamma_{ij}\ell_{ij}^{2}$ in the 2D FG model (see in Equation (4)) and $S_{1}=\sum_{ij}\Gamma_{ij}\ell_{ij}^{2}$ in the 3D FG model (see in Equation (8)) with the effective tensions $\gamma_{ij}$ and $\Gamma_{ij}$, where the sum over triangles $\sum_{\Delta }$ of $S_{1}$ in Equation (4) can be rewritten by using the sum over bonds $\sum_{ij}$. This is the main advantage of the FG model over its canonical counterpart.

Another feature of the FG model is that it is constructed by extending the linear geometry for polymers to a 2D surface or a 3D body. In other words, the FG model is a 2D or 3D extension of Doi–Edwards model for polymers [36]. Indeed, the discrete expressions of $S_{1}$ in Equations (4) and (8) is considered as an extension of the Gaussian chain model, which is mathematically supported by the central limit theorem in probability theory for the variable of the chain extension using the notion of coarse graining [36,53].

### 4.2. Concluding Remarks

We studied the large-strain J-shaped diagrams of biological membranes such as animal skin, muscles, and arteries by 2D and 3D Finsler geometry (FG) models. These materials are very soft, and the zero-stress region of the diagrams ranges from approximately 50% to 150%. Because of this zero-stress region, the diagram is called J-shaped. The J-shaped diagram is roughly composed of two linear lines except the failure region: one is the toe region, and the other is a linear region. The region where these two lines are smoothly connected is called the heel. Based on the word “heel” we can divide the experimental large-toe diagrams into two groups: diagrams with a small heel (S-heel) and diagrams with a large heel (L-heel). The diagrams with an S-heel are consistent with the results of the 2D model, while the diagrams with an L-heel are well fitted by the results of the 3D model. Moreover, the experimental diagrams with a convex part in the large-strain region can be fitted by the 2D FG simulation data. These observations show that the FG modeling technique is applicable for analyzing J-shaped diagrams of biological membranes.

To be more precise, we show that the curve with an S-heel is successfully reproduced by our two-dimensional (2D) FG model, while the curve with L-heel is not described by the 2D FG model but can be reproduced by a 3D FG model. The large strain curves with a convex part are found to be well-fitted by 2D FG model data. Our results in this paper indicate that the FG modeling technique can be used to analyze a wide range of J-shaped stress–strain diagram of biological materials such as tendon, skin, muscles, and arteries. Since the main component that maintains the mechanical strength of these materials is a polymer such as collagen fiber, the polymeric degrees of freedom can be coarse-grained and are simply replaced by the variable *σ*($\in S^{2}$: unit sphere) in the FG model. This simple coarse graining is key to understanding the mechanical properties of these biological materials mathematically.

The fact that the FG model was successfully applied to large-strain J-shaped diagrams indicates that highly non-linear large-strain diagrams of polymers, including those with rubber elasticity, can also be targeted [36,54]. As demonstrated in this paper, FG modeling describes the “strain induced alignment of polymer.” By extending the model slightly or by using 3D body lattices, we can apply the model to large-strain polymeric materials such as rubbers, where the strain-induced crystallization (SIC) plays an important role in their mechanical property [55]. This will be a biology-inspired challenge in understanding new materials and their possible mechanical properties [3,4].

## Figures and Tables

**Table 1:** The parameters assumed for the 2D and 3D models. The units of the parameters are λ[β], κ[β], $H_{0}\left[a\right]\left(=D_{0}\left[a\right]\right)$ and *a*[m], where $\beta =1/k_{B}T$.

	Model	λ	κ	$H_{0}$	*a*
Figure 6a	2D	1	0.6	14	$0.83\times 10^{-9}$
Figure 6b	2D	1	0.55	11.7	$0.85\times 10^{-9}$
Figure 7a	2D	1	0.6	15	$0.52\times 10^{-8}$
	3D	0.4	−0.05	8	$0.75\times 10^{-8}$
Figure 7b	2D	1	0.6	13	$0.17\times 10^{-8}$
	3D	0.45	−0.05	7.6	$0.25\times 10^{-8}$
Figure 8a	2D	1	0.6	15.5	$0.23\times 10^{-8}$
	3D	0.4	−0.05	8.6	$0.34\times 10^{-8}$
Figure 8b	2D	1	0.6	16	$0.23\times 10^{-8}$
	3D	0.4	−0.05	8.6	$0.33\times 10^{-8}$
Figure 9a	2D	1	0.6	13.5	$0.52\times 10^{-8}$
	3D	0.35	−0.1	8.3	$0.83\times 10^{-8}$
Figure 9b	2D	1	0.5	9.8	$0.44\times 10^{-8}$

## Abbreviations

The following abbreviations are used in this manuscript:

2D	2-dimensional
3D	3-dimensional
S-heel	small heel
L-heel	large heel
FG	Finsler geometry
MC	Monte Carlo
